# Low-Parameter Small Convolutional Neural Network Applied to Functional Medical Imaging of Tc-99m Trodat-1 Brain Single-Photon Emission Computed Tomography for Parkinson’s Disease

**DOI:** 10.3390/jpm12010001

**Published:** 2021-12-21

**Authors:** Yu-Chieh Chang, Te-Chun Hsieh, Jui-Cheng Chen, Kuan-Pin Wang, Zong-Kai Hsu, Pak-Ki Chan, Chia-Hung Kao

**Affiliations:** 1 Department of Nuclear Medicine and PET Center, China Medical University Hsinchu Hospital, Hsinchu 302, Taiwan; D25118@mail.cmuhch.org.tw; 2 Department of Nuclear Medicine and PET Center, China Medical University Hospital, Taichung 404, Taiwan; d10119@mail.cmuh.org.tw; 3 Department of Biomedical Imaging and Radiological Science, China Medical University, Taichung 404, Taiwan; 4 Neuroscience Laboratory, Department of Neurology, China Medical University Hospital, Taichung 404, Taiwan; D13132@mail.cmuhch.org.tw; 5 School of Medicine, College of Medicine, China Medical University, Taichung 404, Taiwan; 6 Department of Neurology, China Medical University Hsinchu Hospital, Hsinchu 302, Taiwan; 7 Center of Augmented Intelligence in Healthcare, China Medical University Hospital, Taichung 404, Taiwan; A35374@mail.cmuh.org.tw (K.-P.W.); A27878@mail.cmuh.org.tw (Z.-K.H.); T33768@mail.cmuh.org.tw (P.-K.C.); 8 Graduate Institute of Biomedical Sciences, School of Medicine, College of Medicine, China Medical University, Taichung 404, Taiwan; 9 Department of Bioinformatics and Medical Engineering, Asia University, Taichung 413, Taiwan

**Keywords:** Parkinson’s disease, dopamine transporter, convolutional neural network, Tc-99m Trodat-1 brain single-photon emission computed tomography, deep learning, machine learning, neural network

## Abstract

Parkinson’s disease (PD), a progressive disease that affects movement, is related to dopaminergic neuron degeneration. Tc-99m Trodat-1 brain (TRODAT) single-photon emission computed tomography (SPECT) aids the functional imaging of dopamine transporters and is used for dopaminergic neuron enumeration. Herein, we employed a convolutional neural network to facilitate PD diagnosis through TRODAT SPECT, which is simpler than models such as VGG16 and ResNet50. We retrospectively collected the data of 3188 patients (age range 20–107 years) who underwent TRODAT SPECT between June 2011 and December 2019. We developed a set of functional imaging multiclassification deep learning algorithms suitable for TRODAT SPECT on the basis of the annotations of medical experts. We then applied our self-proposed model and compared its results with those of four other models, including deep and machine learning models. TRODAT SPECT included three images collected from each patient: one presenting the maximum absorption of the metabolic function of the striatum and two adjacent images. An expert physician determined that our model’s accuracy, precision, recall, and F1-score were 0.98, 0.98, 0.98, and 0.98, respectively. Our TRODAT SPECT model provides an objective, more standardized classification correlating to the severity of PD-related diseases, thereby facilitating clinical diagnosis and preventing observer bias.

## 1. Introduction

Parkinson’s disease (PD) is a progressive neurodegenerative disease that affects movement. Its pathological mechanism involves the degeneration of the substantia nigra of the brain stem, which causes the dopaminergic neurons in the basal ganglion to degenerate and the dopamine in the synapses to decrease. This leads to a gradual loss of motor function. The clinical manifestations of PD include tremors, stiffness, bradykinesia, dyskinesia, and impaired posture. Parkinsonism refers to some diseases with symptoms similar to those of PD, but its etiology is not necessarily related to dopaminergic neuron degeneration. Differentiating PD from Parkinsonism is essential in patient treatment and prognosis. However, the objective grading of PD severity is difficult. PD and Parkinsonism are often indistinguishable in structural imaging modalities such as computed tomography (CT) and magnetic resonance imaging (MRI). Thus, functional imaging with a dopamine transporter scan (e.g., Tc-99m Trodat-1 brain (TRODAT) single-photon emission CT (SPECT)) is widely used [1] to determine the number of dopaminergic neurons in the brain as a crucial basis for differential diagnosis.

Functional imaging, a medical imaging technology is used to detect metabolic changes, blood flow, local chemical composition, and nutrient absorption and is extremely different from structural imaging through CT and MRI. High sensitivity and specificity are typical features of functional imaging when no significant changes of the biomarkers or anatomical features are noted. Thus, functional images are more sensitive to blood flow, metabolism, or cell activity in the early stages. Functional imaging has therefore become a crucial diagnostic tool [2]. It mainly examines the physiological activities in specific tissues or organs by using the Digital Imaging and Communications in Medicine (DICOM) format.

Functional imaging typically uses radioactive tracers to reflect the distribution and metabolism in target organs. For instance, radionuclides, which emit radiation, are often used in nuclear medicine for diagnosis. These radionuclides include Tc-99m, Gallium-67, Thallium-201, Iodine-131, etc. and are marked on radioactive tracers taken up by specific tissues, organs, or lesions. However, the contents of these imaging agents are usually similar to certain chemical compounds in the body. For example, in the most common functional imaging modality, positron emission tomography (PET), glucose is used as a typical radioactive tracer [3,4].

In recent years, convolutional neural networks (CNN) have made notable breakthroughs in computer-aided diagnosis based on image recognition [5]. Numerous functional imaging techniques have been integrated with CNNs [6,7,8,9]. CNNs may be applied in functional imaging for clinical diagnosis in hospitals. However, a specific clinical workstation (Intel Core Pentium or i3; 4–8 Gb RAM, 512 Gb–1 Tb HDD) is not designed for deep learning. Therefore, algorithms with an excessive number of parameters or deep layers cannot be used.

This paper presents a model to resolve the aforementioned issue. We developed a set of functional image deep learning algorithms suitable for clinical examination. The proposed model is shallower, more efficient, and more accurate than VGG16, ResNet50, and other popular models. Models with a better efficiency and higher accuracy are more suitable for functional imaging. Our algorithms can label clinical images, which clinicians can then use to classify the severity of Parkinson’s disease by facilitating the diagnosis of TRODAT SPECT. The aim of this paper is to develop a set of functional image deep learning algorithms suitable for a clinical examination. Furthermore, this model utilizes very experienced nuclear medicine physician labeling to help the training of junior physicians and hospitals without the experience for the accurate interpretation of TRODAT SPECT images.

## 2. Materials and Methods

### 2.1. Imaging Data and Equipment

The data of 4305 patients who underwent TRODAT SPECT between June 2011 and December 2019 were analyzed. After the data of patients for whom no images were available were excluded, 3349 pieces of data remained. These data were labeled by five nuclear medicine physicians.

The image values were limited to between 31 and 199 to reduce the image noise and blurring and avoid deviations in the results. We selected only images with a pixel size of 2.209 to prevent edge features that affect the neural network weight due to image space pixel spacing issues, which can ultimately affect the overall image magnification. After the aforementioned conditions were applied, 9564 images of data remained. The male-to-female ratio was approximately 47:53, and the mean age was 66.29 ± 12.79 years (range: 20–107 years).

**Figure 1:**
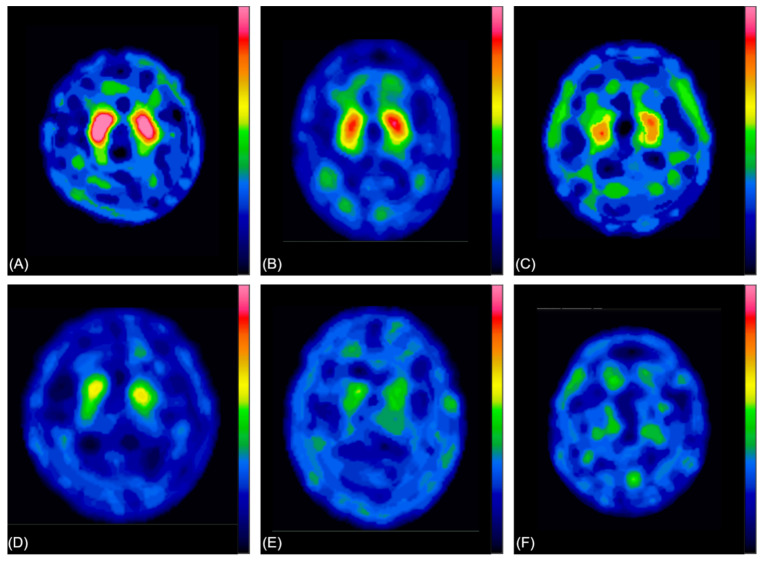
Functional images marked for Parkinson’s disease severity by clinicians. From the top left to the bottom right: categories 0–5 (total: 6 categories). (**A**) category 0, normal striatal uptake bilaterally; (**B**) category 1, normal caudate uptake with putamen uptake loss of >50% on one side and <50% on the other; (**C**) category 2, normal caudate uptake with putamen uptake loss of >50% bilaterally or reduced caudate and putamen uptake of approximately 50% bilaterally; (**D**) category 3, caudate uptake loss of <50% with no putamen uptake; (**E**) category 4, a condition between categories 3 and 5; (**F**) category 5, no striatal uptake bilaterally.

**Figure 2:**
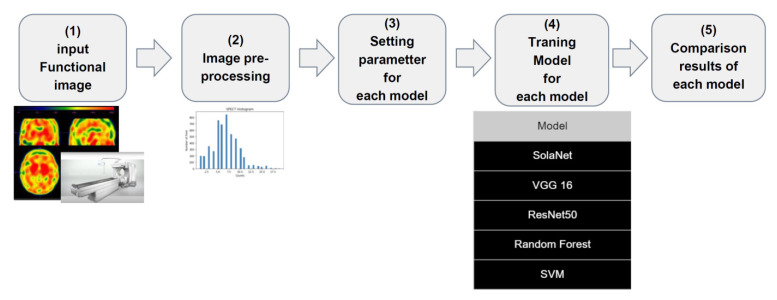
Flow of the application of our small convolutional neural network, which has a low number of parameters, to functional imaging.

All materials used were provided by China Medical University Hospital, including the GE Discovery 670 Pro (GE Healthcare, Chicago, IL, USA), a dual-head SPECT scanner with low-energy reflection collimator (LEHR collimator), and the GE Xeleris 4.0 workstation for image projection. The input image datasets were grayscale images. For each subject, only the maximum active absorption slice of the striatum and other slices, which were the previous slice and the one to the active one, was obtained in order to simulate how to diagnose and determine the disease in the clinic. Therefore, three images collected from each patient were used in the analysis dataset. The total number of images was 9564, and those were merged into 3188 patients and then put into the model. The matrix size was 128 × 128. Two clinicians performed a visual and semiquantitative analysis to categorize the TRODAT SPECT images based on their image characteristics into their “fine visual score” categories proposed by Huang et al. [10]: category 0, normal striatal uptake bilaterally; category 1, normal caudate uptake with putamen uptake loss of >50% on one side and <50% on the other; category 2, normal caudate uptake with putamen uptake loss of >50% bilaterally or reduced caudate and putamen uptake of approximately 50% bilaterally; category 3, caudate uptake loss of <50% with no putamen uptake; category 5, no striatal uptake bilaterally; and category 4, a condition between categories 3 and 5. Figure 1A–F represents the severity of 0–5 for classification. The experimental process is shown in Figure 2.

### 2.2. Computing Equipment

To develop ability training suitable for clinical end computers, the training model was developed on NVIDIA DGX2; after training, the model was implemented on multiple clinical end computers. The DGX2 equipment list is as follows: Dual Intel Xeon Platinum 8168, 2.7 GHz, 24-cores × 2; 1.5 TB RAM; NVIDIA TeslaV100 16 Gb; 512 Gb GPU.

### 2.3. Data Preprocessing

Given that the extent of radiopharmaceutical absorption differs among patients, functional imaging produces distinct tabletop images according to the individual. The changed processing parameters result in varied pixel values in the output images, generating a gap in the images that can affect the judgment of the image characteristics by the model. Therefore, we included image normalization as a preprocessing step.

### 2.4. Models

#### 2.4.1. Model I: SolaNet (Our Proposed Model)

**Figure 3:**
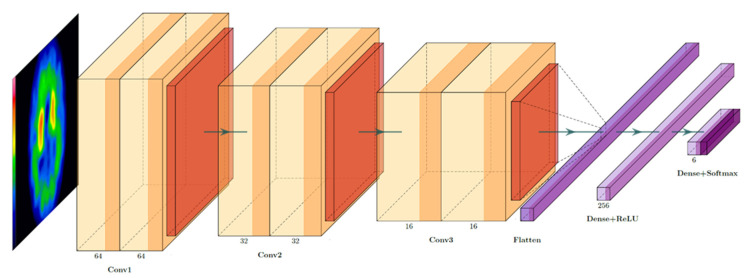
Architecture of SolaNet, our proposed model.

The purpose of this article is to develop a set of functional image deep learning algorithms suitable for clinical examination. Our proposed CNN-based deep learning model for functional images, SolaNet, has fewer layers than Visual Geometry Group (VGG) and fewer parameters than ResNet (Figure 3). It contains several convolutional layers, pooling layers, flatten layers, and dense layers and outputs six categories of prediction results. We employed two convolutional layers to extract features and then converged them through the pooling layer. We also applied maximum pooling instead of average pooling. Considering that functional inspection images do have a large amount of noise and that the image noise causes unstable feature extraction if average pooling is used, maximum pooling was used to ensure that the shape border yielded an excellent image gradient. For the output layer, we applied the normalized exponential function Softmax, because in multiclass labels, the most probable result should be the output. Therefore, for the objective function, we used cross-entropy to classify and calculate the probability of all six categories and then took the highest value as the image prediction result.

#### 2.4.2. Model II: VGG16

AlexNet (developed by researchers at the University of Toronto) and VGGNet (developed by the Visual Geometry Group at Oxford University)—the first runner-up of the ImageNet Large-Scale Visual Recognition Challenge (ILSVRC) 2012 and first runner-up of the ImageNet Large-Scale Visual Recognition Challenge (ILSVRC) 2014 in the classification task, respectively—are two of the most capable object detection models. VGG and AlexNet have similar and comparable performances [11]. VGGNet increases the network depth by reducing the number of convolution kernels, which improves the computing performance. The VGG16 network structure, consisting of only 3 × 3 convolutional layers and 2 × 2 pooling layers, is relatively simple and has high expandability. Replacing fully connected layers, the convolutional layers can adapt to images of various sizes. VGG is a major mainstream model used for image feature extraction.

#### 2.4.3. Model III: ResNet

In 2016, He et al. proposed using residual learning to resolve the deep network degradation problem [12]. The residual neural network (ResNet) is based on the VGG19 network structure, where the size of the feature map is halved but the number of feature maps is doubled. Through shortcut connections, it joins the residual unit, using residual learning to resolve the degradation problem. ResNet was the winner of 1st place in the 2015 ImageNet Large-Scale Visual Recognition Challenge (ILSVRC 2015 (for detection and localization) and COCO 2015 (for detection and segmentation).

#### 2.4.4. Model IV: Random Forest

Random forest (RF), a flexible, easy-to-use supervised machine learning algorithm, produces excellent results in most cases, even without hyperparameter tuning. It is also one of the most used algorithms because of its simplicity and versatility. Specifically, it can be employed in both classification and regression tasks. RF training is a commonly used method in data mining. Through joint decision-making with multiple classification and regression trees and the GINI algorithm, RF randomly allocates training data to reduce the loss of the model [13].

#### 2.4.5. Model V: Support Vector Machine

Support vector machine (SVM), first proposed by Cortes and Vapnik in 1995, has multiple unique advantages in solving small-sample, nonlinear, and high-dimensional pattern recognition problems. It can also be extended to machine learning problems such as function fitting. SVM is a supervised learning model in classification and regression analyses [14]. The SVM model maps data as points in a space to obtain a separate category, such that the categories are as distinct as possible. It then predicts the optimal solution according to the Euclidean distance.

### 2.5. Model Estimation Parameters

To evaluate the performances of all the models, we calculated their accuracy (Equation (1)), precision (Equation (2)), recall (Equation (3)), F1-score (Equation (4)), and area under the receiver operating characteristic (ROC) curve (AUC). The ROC curve is a comprehensive indicator of two continuous variables: specificity and sensitivity. The true positive (sensitivity/true positive) is plotted on the ordinate, and the false positive (specificity/false positive) is plotted on the abscissa as a curve. On the ROC curve, the point closest to the upper left of the graph is the critical point with higher specificity and sensitivity. The AUC is often used as a standard for evaluating the pros and cons of a forecasting model. In other words, the larger the AUC, the more favorable the sensitivity (Equation (5)), specificity (Equation (6)), diagnostic accuracy, and classification performance of a model. If the AUC = 1, the model is perfect. However, in most cases, such perfect models do not exist. By contrast, if the AUC = 0.5, the classification effect of the model is no different from random guessing; in other words, the model has no discriminative ability.

(1) $$Accuracy=\;\frac{TP+TN}{\begin{array}{c} \left(TP+FP+TN+FN\right) \end{array}}$$

(2) $$Precision=\frac{TP}{TP+FP}$$

(3) $$Recall=\frac{TP}{TP+FN}$$

(4) $$F1-score=2*\frac{Recall\;*\;Precision}{Recall+Precision}$$

(5) $$Sensitivity=\frac{TP}{TP+FN}$$

(6) $$Specificity=\frac{TN}{FP+TN}$$

## 3. Results

In this study, we compared SolaNet with four deep and machine learning models—namely, VGG16, ResNet, Random Forest, and SVM—to classify functional images. We included three functional images collected from each patient. One presented the maximum absorption of the metabolic function of the striatum and adjacent two images. Of the 3188 patients, 75%, 15%, and 10% were used as the training, validation, and test sets, respectively (Table 1).

As shown in Table 2, our collected data had different distributions in different categories. To avoid relying on a specific training or test dataset, which could produce bias, we adopted k-fold cross-validation, which can be used for hyperparameter tuning. We first split the training set into k parts. We then took out 1/k parts each time as the validation set and used the rest as the training set. We finally obtained k validation sets. The advantage of k-fold cross-validation is that, even when the amount of training data is low, the generalization ability of the model is tested multiple times on a limited training set, and the results obtained by calculating the average value through multiple validation sets are often more representative.

**Figure 4:**
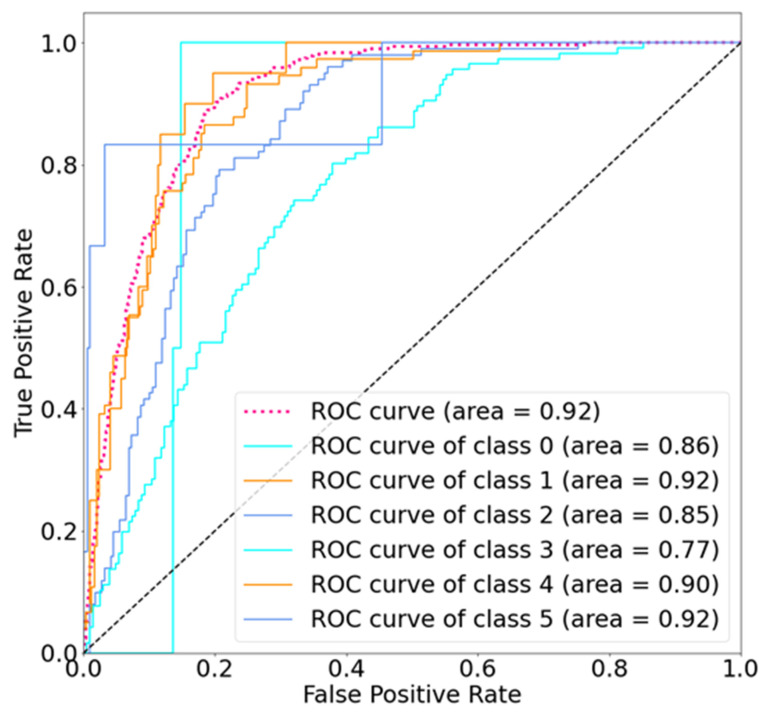
Receiver operating characteristic curve of SolaNet.

Herein, recall refers to the percentage of positive predictions in the total number of positive cases, and precision refers to the number of true positive cases in the total number of positive predictions. If the two evaluation indicators of recall and precision are both high, the results are favorable. The F1-score, a harmonic between recall and accuracy, can be used to roughly evaluate the model performance. Figure 4 demonstrates the high accuracy of SolaNet for functional image inspection, with AUC = 0.92 and favorable classification performance.

As presented in Table 3 and Table 4, SolaNet outperformed the other models in all six categories. For grayscale images, the accuracy, precision, recall, F1-score, and AUC of SolaNet were 0.62, 0.61, 0.62, 0.60, and 0.92, respectively, whereas those of ResNet were 0.58, 0.60, 0.58, 0.56, and 0.89, respectively. In terms of model parameters, SolaNet was only 1 million in size, 25 million lower than VGG and 138 million lower than ResNet. RF and SVM have no comparable parameters; therefore, they could not be included in the comparisons.

In sum, SolaNet is highly accurate. Moreover, the prediction speed and training time of SolaNet are faster and shorter than those of other deep learning networks, respectively.

## 4. Discussion

“Classification of the Multiple Stages of Parkinson’s Disease by a Deep Convolution Neural Network Based on 99mTc-TRODAT-1 SPECT Images” is the CNN model architecture proposed by I-Shou University for TRODAT SPECT classification [15]. It uses five CNN models—namely, AlexNet, GoogLeNet, ResNet, VGG, and DenseNet—for deep learning. This requires 829 PD images, with 70% and 30% of the data used as the training and validation sets, respectively.

In the current study, functional images were collected over 10 years from 3188 patients. These images were merged into one and then put into the model. The real-world data confirmed the clinical excellence of our results.

We next compared the results of SolaNet with those of four deep and machine learning models—namely, VGG16, ResNet, RF, and SVM. SolaNet requires fewer parameters; it is more than 100 times smaller than ResNet50. Such a lightweight model is easier to run on regular hospital computers. Our model is also more efficient than VGG16 and ResNet50.

In the past, VGG16 and ResNet50 had more parameters and deeper network depths, which increased the fitting ability of the neural networks and resulted in more complex expression functions. We believe that the structure and features of functional medical images are not as complex as natural images, so overly complex neural networks may lead to overfitting. In general, we believe that the simpler the neural network, the better the results. Therefore, we propose the SolaNet architecture. The results also demonstrate that neural networks with fewer parameters can outperform previous deep networks in medical image interpretation.

Overall, SolaNet is a shallow, efficient, and accurate model. It considerably reduces the requirement for high-performance computer equipment in hospitals; moreover, it aids auxiliary functional imaging-based diagnosis.

In general, a fine visual score is positively correlated with PD severity and the Hoehn and Yahr (H&Y) stages [16]. However, PD is a progressive disease that sometimes cannot be categorized precisely by such ordinal scales. 

In a multicentered peer review, the interpretation of fine visual scores may vary according to the nuclear medicine physician involved. In general, the acceptable range is from +1 to −1. Therefore, we adjusted the prediction results; summed up the +1 and −1 of the answer as correct; and therefore improved the accuracy, precision, recall, and F1-score of our model.

If a patient has an organic lesion, a history of surgery, or aging-related changes in the cerebrum, this may influence the interpretation of functional images. Nonetheless, 99m-Tc-Trodat-1 fused with MRI may facilitate the indication of relatively precise regions of interest. However, because our patients did not have simultaneous MRI results, we used a magnetic resonance-based TRODAT template alone. Thus, the use of additional labeling techniques to improve the accuracy of our model under these conditions is warranted.

The aim of this paper is to develop a set of functional image deep learning algorithms suitable for clinical examination. We proposed a low-parameter small convolutional neural network (SolaNet) that could be successful for the classification of functional imaging. Our model is lightweight and performs favorably on clinical work. According to the results, SolaNet has better performance than the other models in all six categories when using the labels +1 and −1. For the grayscale images, the accuracy, precision, recall, and F1-score of SolaNet were 0.98, 0.98, 0.98, and 0.98, respectively, while VGG16 were 0.96, 0.95, 0.96, and 0.96, respectively. Additionally, we successfully reduced the parameters of the network and increased the prediction speed. Furthermore, this model utilizes experienced nuclear medicine physician labeling to help the training of novice physicians and new hospitals in the interpretation the TRODAT SPECT images. There were some limitations in this research, such as: (1) the functional images were not well-defined through a numerical standard and could not be easily denoised, (2) the uneven distribution of data at each H&Y stage, and (3) the other limitation of this study was the use of a retrospective research design. In a future study, we will increase the diversity of the image types for other functional images to optimal the classification models for low-parameter small convolutional neural networks (SolaNet).

## 5. Conclusions

We proposed a set of deep learning algorithms for classification of the functional imaging. Our model is lightweight and performs favorably in clinical workstations. It is also shallower, more efficient, and more accurate than popular models such as VGG16 and ResNet50. In sum, our model facilitates clinical diagnosis through functional imaging but without the requirement of high-performance computers. Our TRODAT SPECT model provides an objective, more standardized classification correlating to the severity of PD-related diseases, thereby facilitating clinical diagnosis and preventing observer bias.

## Figures and Tables

**Table 1:** Quantity and proportion of imported model data.

Dataset	Quantity	Proportion
Training data	2390	75%
Validation data	479	15%
Testing data	319	10%

**Table 2:** Types and quantities of imported data.

Severity (Physician Labeling)	Patients
0	19
1	204
2	1010
3	1157
4	739
5	59
Total	3188

**Table 3:** Performance of SolaNet and other popular models.

Model	Categories = 6					
	ACC	Precision	Recall	F1-Score	AUC	Params (Million)
SolaNet	0.62	0.61	0.62	0.6	0.92	1
VGG16	0.56	0.54	0.56	0.54	0.89	25
ResNet50	0.58	0.6	0.58	0.56	0.89	138
Random Forest	0.52	0.52	0.52	0.50	0.51	--
SVM	0.44	0.45	0.44	0.44	0.48	--

**Table 4:** Performance summed up the +1 and −1 of SolaNet and other popular models.

Model	Category = 6 Label + −1			
	ACC	Precision	Recall	F1-Score
SolaNet	0.98	0.98	0.98	0.98
VGG16	0.96	0.95	0.96	0.96
ResNet50	0.96	0.95	0.96	0.95
Random Forest	0.97	0.96	0.97	0.96
SVM	0.93	0.93	0.93	0.93

## Data Availability

Not applicable.
